# Scrub Typhus Is an Under-recognized Cause of Acute Febrile Illness with Acute Kidney Injury in India

**DOI:** 10.1371/journal.pntd.0002605

**Published:** 2014-01-30

**Authors:** Vivek Kumar, Vinod Kumar, Ashok K. Yadav, Sreenivasa Iyengar, Ashish Bhalla, Navneet Sharma, Ritesh Aggarwal, Sanjay Jain, Vivekanand Jha

**Affiliations:** 1 Departments of Nephrology, Postgraduate Institute of Medical Education and Research, Chandigarh, India; 2 Internal Medicine, Postgraduate Institute of Medical Education and Research, Chandigarh, India; 3 Pulmonary and Critical Care Medicine, Postgraduate Institute of Medical Education and Research, Chandigarh, India; 4 George Institute for Global Health, New Delhi, India; Mahidol University, Thailand

## Abstract

**Background:**

Infection-related acute kidney injury (AKI) is an important preventable cause of morbidity and mortality in the tropical region. The prevalence and outcome of kidney involvement, especially AKI, in scrub typhus is not known. We investigated all patients with undiagnosed fever and multisystem involvement for scrub typhus and present the pattern of renal involvement seen.

**Methods:**

From September 2011 to November 2012, blood samples of all the patients with unexplained acute febrile illness and/or varying organ involvement were evaluated for evidence of scrub typhus. A confirmed case of scrub typhus was defined as one with detectable *Orientia tsutsugamushi* deoxyribonucleic acid (DNA) in patient's blood sample by nested polymerase chain reaction (PCR) targeting the gene encoding 56-kDa antigen and without any alternative etiological diagnosis. Renal involvement was defined by demonstration of abnormal urinalysis and/or reduced glomerular filtration rate. AKI was defined as per Kidney Disease: Improving Global Outcomes (KDIGO) definition.

**Results:**

Out of 201 patients tested during this period, 49 were positive by nested PCR for scrub typhus. Mean age of study population was 34.1±14.4 (range 11–65) years. Majority were males and a seasonal trend was evident with most cases following the rainy season. Overall, renal abnormalities were seen in 82% patients, 53% of patients had AKI (stage 1, 2 and 3 in 10%, 8% and 35%, respectively). The urinalysis was abnormal in 61%, with dipstick positive albuminuria (55%) and microscopic hematuria (16%) being most common. Acute respiratory distress syndrome (ARDS) and shock were seen in 57% and 16% of patients, respectively. Hyperbilirubinemia was associated with AKI (p = 0.013). A total of 8 patients (including three with dialysis dependent AKI) expired whereas rest all made uneventful recovery. Jaundice, oliguria, ARDS and AKI were associated with mortality. However, after multivariate analysis, only oliguric AKI remained a significant predictor of mortality (p = 0.002).

**Conclusions:**

Scrub typhus was diagnosed in 24% of patients presenting with unexplained febrile illness according to a strict case definition not previously used in this region. Renal abnormalities were seen in almost 82% of all patients with evidence of AKI in 53%. Our finding is contrary to current perception that scrub typhus rarely causes renal dysfunction. We suggest that all patients with unexplained febrile illness be investigated for scrub typhus and AKI looked for in scrub typhus patients.

## Author Summary

A large number of patients present to Indian hospitals with acute febrile illness and multisystem involvement. Acute kidney injury is an important component. Clinical manifestations do not allow distinction between infectious etiologies with overlapping presentations. Many of the cases remain undiagnosed and therefore are not treated, resulting in high mortality. Despite having all the elements of endemicity, scrub tuphus, caused by the rickettsia and transmitted to humans by the bites of trombiculid mite, is not regognized in Indian hospitals due to non-availability of locally validated diagnostic tests. Previous reports have been limited to serologyic testing, with high false positives due to past exposure rather than acute infection. Moreover, kidney involvement in this condition is not well known. We looked for scrub typhus DNA in 201 patients with an acute febrile illness. About 25% tested positive; kidney involvement was seen in over 80%, and acute kidney injury in 53%. Acute kidney injury was a predictor of mortality. All these are new findings of public health importance and suggest the need to include testing for this condition in the diagnostic armanetarium and development of local serological cutoffs.

## Introduction

Infections are responsible for a substantial portion of community acquired acute kidney injury (AKI) in India. The commonly implicated conditions include malaria, leptospirosis, dengue, enteric fever, viral and bacterial infections. Despite being endemic in Asia with an estimated one million cases occurring annually, scrub typhus, caused by the rickettsia *Orientia tsutsugamushi*, is highly underdiagnosed and under-reported cause of hospitalization [1], [2]. World Health Organization (WHO) identifies scrub typhus as a re-emerging disease in South-East Asia and the South-Western Pacific region with a case fatality rate of up to 30% in untreated cases and stresses the need for its surveillance [1]. Scrub typhus has been reported from various parts of India [3]–[8], and has recently been identified as one of the important neglected zoonoses of public health importance [9].

Scrub typhus is considered as an uncommon cause of AKI even in endemic areas. Renal involvement is thought to be a consequence of multi-organ dysfunction syndrome secondary to sepsis [10], [11]. In part, this has been due to inability to make an accurate diagnosis due to non-availability of investigations. Recent studies have suggested a higher prevalence of kidney involvement in this condition. These studies, however, have used either clinical features alone or in combination with serology with single sample arbitrary antibody titre cut offs [12] to make a diagnosis. Reliability on serology alone is problematic in endemic areas, and use of nucleic acid based testing (NAT) is recommended [13]–[15].

We prospectively studied the pattern of kidney involvement and its impact on the outcome in scrub typhus patients who were diagnosed by using a strict NAT based case definition.

## Materials and Methods

### Ethics statement

The study was approved by the Institute Ethics Committee of the Postgraduate Institute of Medical Education and Research. Written informed consent was obtained from all adult patients and from the parents or legal guardians of minor subjects. The study was conducted in accordance to the principles of the Declaration of Helsinki.

### Study setting and subjects

This study was done at the Nehru Hospital of Postgraduate Institute of Medical Education and Research, the largest tertiary care referral hospital in North India located near the foothills of The Great Himalayas. From September 2011 to November 2012, all patients referred to our hospital with unexplained fever and/or varying degrees of multisystem involvement were tested for *O. tsutsugamushi* deoxyribonucleic acid (DNA) in blood by nested polymerase chain reaction (PCR) targeting gene encoding for the 56-kDa antigen of Gilliam strain of *O. tsutsugamushi* [16].

All patients also underwent at least three blood cultures for bacterial growth, three peripheral blood film examinations for malarial parasite and malarial antigen detection in blood by immunochromatographic rapid card test (QDx Malaria PAN/Pf and Pv/Pf, Piramal Healthcare, India). In addition, dengue virus NS1 antigen, dengue virus IgM antibody and leptospiral IgM antibody were tested in blood samples of all patients.

Demographic details and clinical course were recorded for all patients. A detailed history which included recording of signs and symptoms, occupational details, geographic and social background, previous co-morbidities and treatment was taken and complete physical examination was done in all patients to specifically look for presence of eschar, rash and lymphadenopathy. All routine hematological and biochemistry profiles were noted at admission and follow up. Urine was tested using dipstick for albumin and sugar, and microscopic examination of freshly voided urine specimen was performed in all patients. Hourly urine output and daily serum creatinine monitoring were done during hospital stay. Complications were noted for all patients.

### Variables

A confirmed case of scrub typhus was defined as the one with positive nested PCR for *O. tsutsugamushi* with no other evidence of any other infection. As single point qualitative immunoglobulin M (IgM) enzyme linked immunosorbent assay (ELISA) has been extensively used to define cases of scrub typhus in published literature especially from our region where the disease appears to be highly endemic, scrub typhus IgM antibody was also looked for in patients' sera by ELISA in all cases by commercially available qualitative ELISA kit (InBios International, Inc., USA) according to the manufacturer's instructions. The test was considered to be positive if the antibody was present at dilution of ≥1∶100 as defined by the manufacturer.

Shock was defined as systolic blood pressure <90 mm Hg or requirement of inotropes; acute respiratory distress syndrome (ARDS) defined as PaO2/FiO2≤300 mm Hg [17]; severe anemia as hemoglobin <9 g/dL; leukocytosis as total leukocyte count >11000 cells/µL; leukopenia as total leukocyte count <4000 cells/µL; thrombocytopenia as platelet count <100000 cells/µL; hypoalbuminemia as serum albumin <3.5 g/dL; hyperbilirubinemia as serum total bilirubin >1.2 mg/dL; elevated serum glutamate oxaloacetate aminotransferase (SGOT) as >40 U/L; elevated serum glutamate pyruvate aminotransferase (SGPT) as >40 U/L and elevated serum alkaline phosphatase (SAP) as >130 U/L.

AKI was defined and staged according to Kidney Disease: Improving Global Outcomes (KDIGO) definition [18].

### Polymerase chain reaction for 56-kDa antigen of of *O. tsutsugamushi*

**Figure 1:**
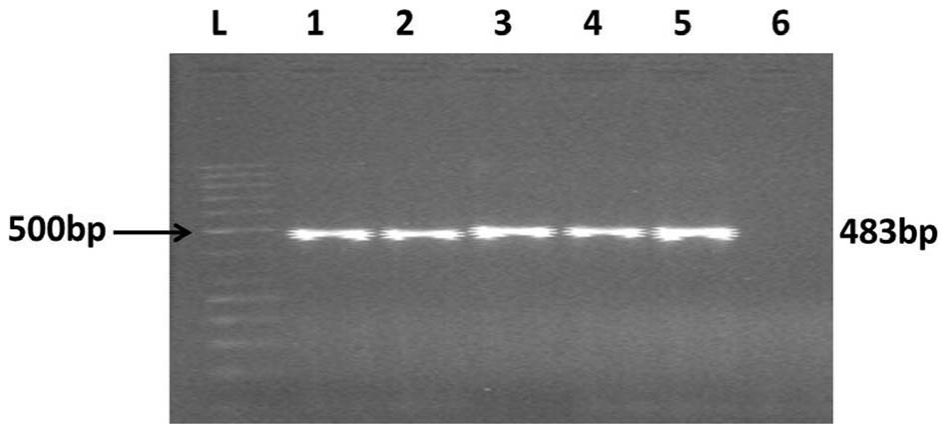
The ethidium bromide-stained agarose gel shows the 483 bp polymerase chain reaction products. L: 500 bp ladder, 1–4: patients' samples; 5: positive control; 6: negative control.

**Figure 2:**
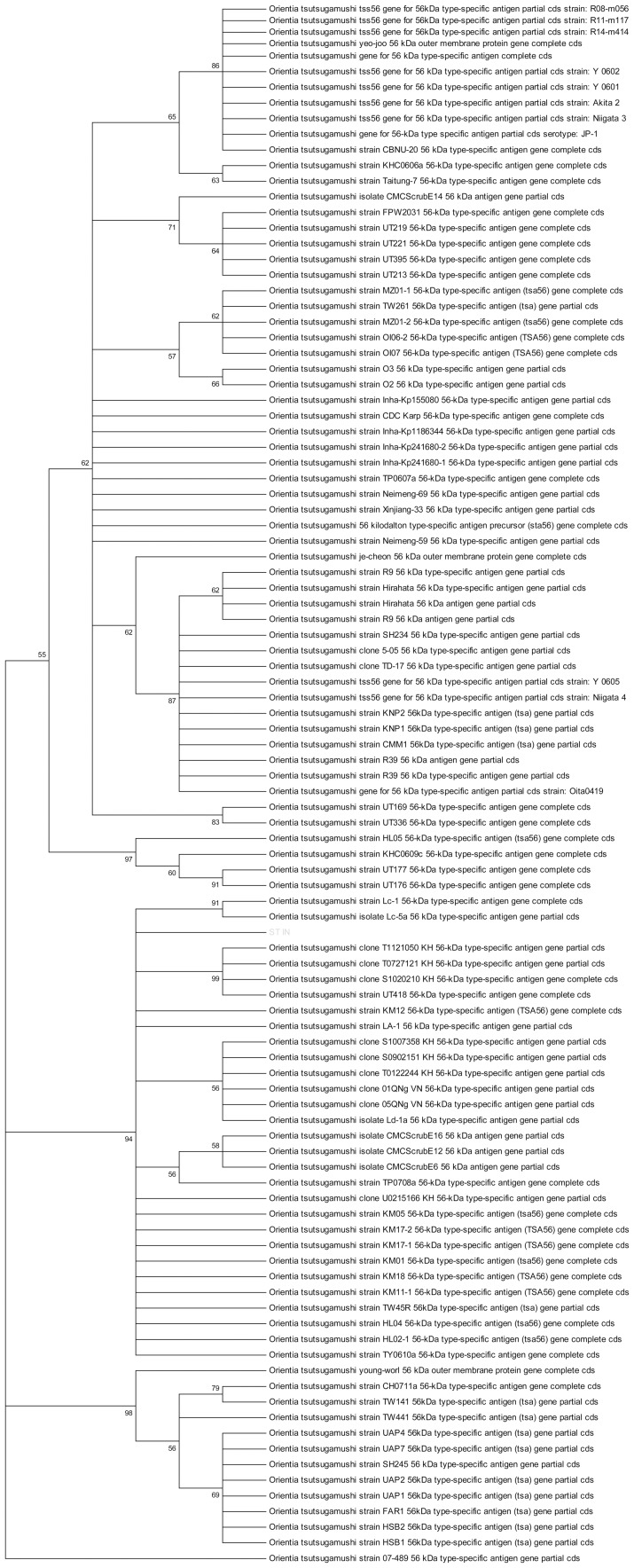
Phylogenetic tree analysis, highlighted test sample shows >98% homology to *O. tsutsugamushi*.

Genomic DNA was isolated from buffy coat of 5 ml blood collected in ethylenediaminetetraacetic acid (EDTA) vial using QIA amp DNA mini kit according to manufacturer's instructions (Qiagen. Hilden, Germany). Nucleotide specific sequence amplification was done for the presence of 56-kDa antigen of of *O. tsutsugamushi*. A set of nested PCR primers were used for two rounds of amplification (outer primers, forward: TCAAGCTTATTGCTAGTGCAATGTCTGC, reverse: AGGGATCCCTGCTGCTGTGCTTGCTGCG; inner primers, forward: GATCAAGCTTCCTCAGCCTACTATAATGCC, reverse: CTAGGGATCCCGACAGATGCACTATTAGGC) using conditions described earlier [16], [19]. Visualization of specific 483 bp amplification product was considered to be diagnostic (Figure 1). A previously confirmed scrub typhus patient sample (fever with eschar, ≥4 fold rise in IgM antibody titres by enzyme linked immunosorbent assay and positive nested PCR for gene encoding 56-kDa antigen of *O. tsutsugamushi* confirmed by sequencing) was taken as positive control. For each run, one positive human control, one healthy negative human control and one negative control (without any DNA) were run simultaneously to confirm the accuracy. Sequencing of PCR product was done in 3 randomly selected patients. Sequence alignment was done with nucleotide blast on pubmed and analyzed using Molecular Evolutionary Genetics Analysis (MEGA) software for phylogenetic tree analysis. The results showed >98% homology with *O. tsutsugamushi* family strains (Figure 2, test sequence highlighted). This nested PCR was also performed on eschar tissue scraped from 2 patients who had positive blood samples.

### Management

The patients were treated with either doxycycline (100 mg twice daily for 7 days) or azithromycin (500 mg daily for 3 days). Fluid overload, hyperkalemia or metabolic acidosis refractory to medical measures, or persistent oliguria were indications for starting dialysis.

### Outcomes

Urinalysis abnormalities and AKI were the outcomes of interest. The study population was divided into two groups based on presence or absence of AKI.

### Statistical analysis

Data was analysed using Statistical Package for the Social Sciences (SPSS) software for Windows, version 20.0 (IBM Corp., Armonk NY). Univariate analysis was done to assess association of various parameters with presence of AKI and final outcome. Binary logistic regression analysis (forward conditional) was used to look for independent association of clinical and laboratory factors with the outcome. All p values were two-tailed, and considered to be significant at <0.05.

## Results

Out of 201 patients with fever and multisystem involvement seen during this period, 49 tested positive for *O. tsutsugamushi* DNA. None of these had any alternative diagnosis (Table 1). Amongst 152 patients who were negative for scrub typhus by aforementioned PCR, 20% patients had an alternative diagnosis (Table 2). Importantly, a total of 105 patients were recorded as positive for scrub typhus IgM ELISA. These included 43% of scrub typhus PCR negative patients. A total of 23% of patients within this subset (scrub typhus PCR negative but IgM ELISA positive) were assigned an alternative diagnosis. A comparison of the clinical features and laboratory abnormalities in scrub typhus positive and negative patients is given in Table 1. Vomiting, altered sensorium, bodyaches, tachycardia, hepatomegaly, lymphadenopathy, AKI and thrombocytopenia were significantly more common in patients with scrub typhus (Table 1).

**Table 1:** Clinical and laboratory manifestations in patients (total no. of patients: 201).

Parameter	Scrub typhus (n = 49)	Others (n = 152)	P value
**Symptoms**			
Fever	48	151	0.396
Shortness of breath	33	80	0.071
Jaundice	21	50	0.205
Pain abdomen	15	47	0.968
Vomiting	15	18	**0.002**
Decreased urine output	13	36	0.687
Headache	14	37	0.554
Altered sensorium	12	16	**0.014**
Bleeding manifestations	10	16	0.073
Loose motions	5	12	0.613
Bodyaches	3	1	**0.017**
Seizures	2	16	0.169
**Signs**			
Tachycardia	36	54	**<0.001**
Hepatomegaly	34	77	**0.022**
Tachypnea	33	87	0.210
Splenomegaly	20	49	0.271
Lymphadenopathy	10	7	**0.002**
Eschar	9	2	**<0.001**
Rash	2	20	0.077
**Complications**			
Shock	8	12	0.086
ARDS	28	75	0.342
AKI	26	52	**0.020**
Mortality	8	4	**<0.001**
**Investigations**			
Hemoglobin (g/dL)	10.3±2.5	9.9±2.5	0.381
TLC (per µL)	11667±6539	11702±8813	0.980
Platelet count (per µL)	84,804±71,786	129,504±117,721	**0.016**
Serum total bilirubin (mg/dL)	4.9±7.9	3.6±6.0	0.251
SGOT (U/L)	378±1299	190±317	0.149
SGPT (U/L)	160±408	133±189	0.577
SAP (U/L)	279±258	308±334	0.623
Total protein (g/dL)	6.2±1.1	6.1±0.9	0.577
Serum albumin (g/dL)	3.0±0.7	3.0±0.7	0.886
Serum creatinine (mg/dL)	1.9±1.5	1.9±2.3	0.910
Scrub typhus IgM ELISA positivity	39	66	**<0.001**

AKI: acute kidney injury, ARDS: acute respiratory distress syndrome, ELISA: enzyme linked immunosorbent assay, IgM: immunoglobulin M, PCR: polymerase chain reaction, SAP: serum alkaline phosphatase SGOT: serum glutamate oxaloacetate aminotransferase, SGPT: serum glutamate pyruvate aminotransferase, TLC: total leukocyte count.

**Table 2:** Diagnoses in scrub typhus PCR negative population.

	Scrub typhus IgM ELISA		
Diagnosis	Positive	Negative	Total
No of cases	66	86	152
Unknown	51	70	121
Malaria	5	5	10
Dengue	4	3	7
Pyogenic meningitis	2	0	2
Hepatitis A	1	1	2
Hepatitis E	1	0	1
Japanese encephalitis	1	0	1
Leptospirosis	0	1	1
Tuberculosis	0	1	1
Others	1*	5#	6

Drug Reaction with Eosinophila and Systemic Symptoms (DRESS);

^#^ Still's disease, erythema nodosum, polymyositis, dapsone syndrome, thrombotic microangiopathy.

ELISA: enzyme linked immunosorbent assay, IgM: immunoglobulin M, PCR: polymerase chain reaction.

Males constituted 59% of the scrub typhus PCR positive cases. The mean age was 34.1±14.4 (range 11–65) years and the duration of symptoms at presentation was 10.8±4.9 (range 3–30) days. About 84% patients were from rural areas. No cases were diagnosed during summer months from March to June. Majority of the cases occurred after rainy season in July–August and continued through winter.

Table 3 lists the clinical manifestations recorded in this study population. In addition to fever, the main symptoms were shortness of breath, jaundice, pain abdomen, vomiting, decreased urine output, headache and altered mentation. Tachycardia, hepatomegaly, tachypnea and splenomegaly were noted in 73%, 69%, 67% and 41% of the patients, respectively. Eschar was noted in 9 cases; the location being chest (3) and nape of neck, arm, index finger of hand, hypogastric region, gluteal region and popliteal fossa. Amongst these confirmed cases, IgM ELISA for scrub typhus was positive in 80%.

**Table 3:** Comparison of clinical, laboratory manifestations and complications in the AKI and non-AKI groups in the confirmed scrub typhus study population (scrub typhus PCR positive, no. of patients: 49).

Parameter	AKI (n = 26)	No AKI (n = 23)	P value (two tailed)
**Symptoms**			
Fever	26	22	0.283
Shortness of breath	20	13	0.129
Jaundice	16	5	**0.005**
Pain abdomen	6	9	0.224
Vomiting	7	8	0.551
Oliguria	13	0	**<0.001**
Headache	7	7	0.786
Altered sensorium	8	4	0.277
Bleeding manifestations	5	5	0.828
Loose motions	2	3	0.537
Bodyaches	3	0	0.093
Seizures	1	1	0.929
**Signs**			
Tachycardia	20	16	0.560
Hepatomegaly	18	16	0.980
Tachypnea	18	15	0.765
Splenomegaly	13	7	0.164
Lymphadenopathy	4	6	0.354
Eschar	5	4	0.868
Rash	1	1	0.929
**Others**			
Shock	6	2	0.174
ARDS	17	11	0.215
Mortality	8	0	**0.004**
**Investigations**			
Hemoglobin (g/dL)	10.7±2.8	9.7±2.2	0.174
TLC (per µL)	11923±6750	11363±6426	0.771
Platelet count (per µL)	82560±75525	87476±68821	0.820
Serum total bilirubin (mg/dL)	7.6±9.7	1.8±2.9	**0.013**
SGOT (U/L)	587±1742	124±96	0.255
SGPT (U/L)	226±538	75±45	0.243
SAP (U/L)	336±318	217±158	0.152
Total protein (g/dL)	6.2±1.2	6.2±1.0	0.848
Serum albumin (g/dL)	2.8±0.6	3.1±0.8	0.387
Blood urea (mg/dL)	137±68	44±29	**<0.000**
Serum creatinine (mg/dL)	2.8±1.5	0.8±0.2	**<0.000**
Scrub typhus IgM ELISA positivity	25	14	**0.002**
**Complication**			
Severe anemia	8	7	0.980
Leukocytosis	10	8	0.881
Leukopenia	0	1	0.272
Thrombocytopenia	17	15	0.801
Hypoalbuminemia	18	15	0.817
Hyperbilirubinemia	19	7	**0.002**
Elevated SGOT	20	15	0.488
Elevated SGPT	18	11	0.231
Elevated SAP	14	11	0.431

AKI: acute kidney injury, ARDS: acute respiratory distress syndrome, ELISA: enzyme linked immunosorbent assay, IgM: immunoglobulin M, PCR: polymerase chain reaction, SAP: serum alkaline phosphatase SGOT: serum glutamate oxaloacetate aminotransferase, SGPT: serum glutamate pyruvate aminotransferase, TLC: total leukocyte count.

Renal abnormalities were noted in 82% of the scrub typhus patients (Table 4). Abnormal urinalysis was present in 61%, the major abnormalities being dipstick positive albuminuria (55%) and microscopic hematuria (16%). AKI, as defined according to KDIGO definition, was seen in 53% of patients. A total of 5 (10%) patients were in stage 1, 4 (8%) in stage 2 and 17 (35%) in stage 3. Jaundice, oliguria and mortality were significantly more in patients with AKI (Table 3). About half of all patients with AKI were oliguric. There were no significant differences between the oliguric and non-oliguric scrub typhus patients with AKI except for mortality which was significantly higher in oliguric patients (p = 0.011). Gross, painless hematuria was seen in two patients, and one had intravascular hemolysis. A 14-year-old male who presented with fever and gross hematuria underwent a kidney biopsy that showed mesangial hypercellularity with 2+ mesangial deposition of immunoglobulin A (IgA). He continued to have microscopic hematuria after 6 months. One patient had nephrotic range proteinuria at presentation that rapidly improved over 2 weeks and hence, biopsy was not done. Three patients required dialysis.

**Table 4:** Renal abnormalities in the confirmed scrub typhus study population (no. of patients: 49).

Parameter	No. of patients (%)
Oliguria (<400 ml/d)	13 (26)
Gross hematuria	2 (4)
Dysuria	1 (2)
Hemoglobinuria	1 (2)
Abnormal urinalysis	30 (61)
Dipstick positive albuminuria	27 (55)
• ≤2+	26 (53)
• >2+	1 (2)
Dipstick positive glucosuria	1 (2)
Significant pyuria (>9 pus cells/hpf)	3 (6)
Microscopic hematuria (>2 RBC/hpf)#	8 (16)
AKI$	26 (53)
Stage 1	5 (10)
Stage 2	4 (8)
Stage 3	17 (35)

^#^ excludes two patients with gross hematuria;

defined according to KDIGO guidelines for AKI.

AKI: acute kidney injury, KDIGO: Kidney Disease: Improving Global Outcomes, RBC: red blood cell.

Prominent hematological abnormalities included thrombocytopenia, leucocytosis and severe anemia (Table 3). Hyperbilirubinemia, transaminitis, and hypoalbuminemia were also seen in majority of patients. Over 50% patients developed ARDS. Secondary hemophagocytosis and septic arthritis were seen in one patient each. Interestingly, two of the patients were kidney transplant recipients.

Patients with characteristic eschar were immediately started on treatment once blood samples were collected for testing. Otherwise, specific treatment for scrub typhus was started only after diagnosis was confirmed. The disease was fatal in 8 patients, all of whom had ARDS and AKI. All three patients who required dialysis expired. On univariate analysis, jaundice, oliguria, elevated bilirubin, elevated SGOT, elevated SGPT, ARDS and AKI were significantly associated with mortality (p of 0.045, <0.0001, 0.005, 0.022, 0.039, 0.007 and 0.004, respectively). There was no significant association of stage of AKI with mortality (p = 0.214). On multivariate analysis, only oliguric AKI showed an independent association with mortality (p = 0.002, Cox & Snell R Square 0.309).

Except the person with persistent hematuria, all surviving patients had normal renal function and normal urinalysis at 3 months after recovery.

## Discussion

This is the first report to comprehensively document the clinical picture, investigative profile, pattern of renal involvement and outcome in a cohort of patients of scrub typhus using a strict case definition. We show that scrub typhus is responsible for about 24% of all patients presenting with unexplained febrile illness and/or multi-system involvement. We also show that the renal involvement is fairly common in scrub typhus. AKI is seen in over 50% cases, and is an important predictor of mortality.

Scrub typhus is caused by obligate, intracellular bacterium *O. tsutsugamushi* which is maintained in nature by trans-ovarian transmission in trombiculid mites. Human involvement occurs accidentally when they get bitten by infected trombiculid mite larvae (chiggers) leading to inoculation of organisms into skin. Vascular endothelial cell injury leading to vasculitis is the predominant mechanism of cellular injury in this disease.

Scrub typhus does not find a mention in most descriptions of tropical community acquired AKI. In part, the reason has been inability to make an accurate diagnosis. The clinical description lays emphasis on the presence of an eschar, which is often difficult to see in dark-skinned persons. Supporting tests include either demonstration of rising antibody titre or rickettsial DNA [15]. Serologic tests are plagued by under-reporting of methodology and variable seropositivity criteria. [15], [20]. Most Indian reports have relied on a single point commercially available IgM ELISA using cutoffs that have been derived from low endemicity areas. A major limiting factor is lack of availability of local seroprevalence dats. Single time point estimations should be used only when robust local seroprevalence data is available [14], [20]. Variable single point titres in India (≥1∶100, ≥16 units) and Thailand (≥1∶400) have been reported as positive [6], [8], [13]. However, using serology alone, Paris et al used an admission IgM titer of ≥1∶12,800 (based on local seroprevalence data and highest titres seen during acute infections) or a four-fold rise in IgM antibody by immunofluorescence antibody assay (IFA) to define a case of scrub typhus in Thailand [21]. Therefore, use of single sample titres for making a positive diagnosis using arbitrary cutoff without local validation is strongly discouraged [15], [22], [23]. Unfortunately, seroprevalence data in Indian population is not available. Our study shows that diagnosis of scrub typhus using single time qualitative IgM antibody estimation by the commercially available IgM ELISA kit would have failed to diagnose scrub typhus in 20% of PCR positive confirmed cases.

Nested PCR targeting gene encoding for the 56-kDa antigen of Gilliam strain of *O. tsutsugamushi* has been shown to have high sensitivity and specificity. This primer set has been shown to amplify DNA sequences from other strains of *O. tsutsugamushi* [13], [16], [24], [25], and has been suggested as a group specific test for *O. tsutsugamushi* [13]. Importantly, this PCR has been shown to be negative in normal humans and other infections. It is important to note that the serotype specific primers are different from this set and have been described in literature [16]. In a study of 36 patients who presented with fever at a large provincial hospital in Southern Thailand, nested PCR for gene encoding the 56 kDa antigen of Gilliam strain of *O. tsutsugamushi* was compared with IFA (both acute and convalescent sera in only 17 patients) [13]. This PCR detected all 9 patients who were diagnosed by serology (positive if single point titre of >1∶400 or a four-fold rise in titre to at least 1∶200), and in addition was positive in 3 more patients who were negative by serology. The reported range for days after onset of disease and specific drug treatment when samples were taken for testing ranged from 5–33 days and 1–27 days, respectively. Importantly, this PCR was negative in healthy human controls and murine typhus patients. Also, the investigators showed that this PCR amplified DNA of 9 other strains of *O. tsutsugamushi* prevalent in South-East Asia and suggested that it could be used to detect diverse antigenic types of *O. tsutsugamushi*. In a study from Korea, nested PCR for gene encoding the 56 kDa antigen of *O. tsutsugamushi* was shown to have a sensitivity of 82.2% and a specificity of 100% when compared with IFA (diagnosis made by either single point titres cut off or four fold rise in titres in convalescent sera) [26]. In another recent study from the same centre where the investigators compared various types of PCR (47 kDa gene, 56 kDa gene) for scrub typhus diagnosis (confirmed only by four fold rise in convalescent sera titres by IFA), 56 kDa nested PCR was found to have the highest sensitivity (87.8%) and 100% specificity [14]. IFA which is considered current gold standard reference diagnostic method is imperfect, gives retrospective diagnosis and plagued by variability in methodology and arbitrarily defined cut off titres without supporting epidemiological data [23]. Theoretically, PCR based assays would offer diagnosis till bacteremia persists, before antibody response occurs in early phase of disease and overcome the problem of high background titres in endemic areas before antibody response starts appearing. However, once bacteremia has cleared with or without treatment, PCR based assays would be negative. Therefore, it has been suggested that future diagnostic development should preferably focus on both pathogen and antibody based tests [23]. Recently, a set of robust set of reference criteria comprising culture, serological cut offs backed by local epidemiological data and combination of various available PCRs has been proposed for validation of new diagnostic techniques for scrub typhus [21].

Therefore, our study appropriately highlights the current limitations of using such serologic tests for diagnosis of scrub typhus and suggests the need to obtain robust population data to determine appropriate cutoffs for diagnosis. Antibody titre estimation in paired serum samples may overcome these limitations, but the diagnosis would be retrospective in such a situation and may not help in immediately guiding treatment.

Using this nested PCR as confirmatory diagnostic criterion, 24% of patients with unexplained febrile illness seen at our centre had scrub typhus. In a study from South India [27], about 51% of all patients with febrile illness were diagnosed as scrub typhus on the basis of ELISA. It is important to note that there is no data from Indian population which validates antibody titre cutoffs put forward by manufacturer's in Western countries. Had we also based the diagnosis on serology, the prevalence would have been 57%. We did not note any geographic clustering. The condition was also seen in urban residents,who are traditionally considered to be at low risk [28], [29]. Whether this is related to environment, host, pathogen or vector related factors needs further studies. Epidemiological investigation of a recent outbreak of scrub typhus in North-East India suggested emergence of a new species of vector trombiculid mite, *Schoengastiella ligula* [30]. The increased incidence after rainy season is expected as increased vegetation after rains support growth of larvae of trombiculid mite, reflecting tropical disease epidemiology. There is a trend towards more use of fluoroquinolone, cephalosporins, extended spectrum penicillins and newer antibiotics in the community [31], [32]. We believe that increasing use of these antibiotics for treatment of febrile illnesses in the community during recent times may be contributing to unmasking of this disease as the causative organism is inherently resistant to them.

Till recently, renal involvement due to scrub typhus had not received much attention. A recent systematic review could only find a few case reports specifically describing acute renal failure due to scrub typhus [33]. Overall, renal involvement is considered to be a part of multi-organ dysfunction syndrome in patients with severe disease [10], [11]. In Taiwan, incidence of AKI in scrub typhus was reported to be 8.3% and 6.6% in two series [34], [35]. Importantly, clinical course in 8 out of 9 patients who developed acute renal failure in one of these series was complicated by septic shock which might have precipitated AKI [34]. In India, two series from South India have shown the incidence of AKI to be 19% and 42% (Table 5) [6], [27], [36], [37]. A recent series from North India reported AKI in 34% patients [8]. It is important to note that that the basis of diagnosis has been a single point measurement of antibody using an arbitrary cutoff, and therefore subject to the above mentioned limitations. Our study is the first to use a robust diagnostic parameter to identify definitive cases and evaluate renal involvement.

**Table 5:** Renal involvement in scrub typhus.

Study	Diagnostic criteria	Region	No. of patients, age of patients	Urinalysis abnormalities	AKI definition	AKI	Any other form of renal involvement
Kumar M, 2012 [37]	Clinical and Weil Felix	South India	35, age ≤12 years	Not reported	AKIN	20% of all patients (stages 1, 2 and 3 reported as 29%, 42% and 29% of all patients with AKI, respectively)	Oliguria: 43% of all patients
Basu G, 2011 [27]	ELISA	South India	188, age ≥18 years	Not reported	RIFLE	42.6% of all patients (risk, injury and failure in 48%, 26% and 26% of all patients with AKI, respectively)	
Chrispal A, 2010 [6]	IgM ELISA	South India	189, age ≥16 years	Not reported	Serum creatinine >1.4 mg/dL	19.6% of all patients	Oliguria: 10.6% of all patients
Attur RP, 2013 [36]	Weil Felix or IgM ELISA	South-West India	259, median age 39 years	56.7% of all patients	RIFLE	23.2% of all patients (risk, injury and failure in 38%, 22% and 40% of all patients with AKI, respectively)	
Vikrant S, 2013 [8]	IgM ELISA	North India	515, only data about patients with AKI presented (mean age 41.4±15.9 years)	Not reported	RIFLE	35% of all patients (risk, injury and failure in 45%, 34% and 21% of all patients with AKI, respectively)	
Wu KM, 2009 [34]	IFA or PCR	Eastern Taiwan	136, age ≥2 years	Not reported	≥0.5 mg/dL increase in serum creatinine over baseline or lowest value OR 24 hour urine volume <400 ml	6.6% of all patients	
Berman SJ, 1973 [38]	IFA or mouse inoculation*	South Vietnam	87, adults	20% of all patients	Elevated blood urea nitrogen	None	

Used ≥4 fold rise in antibody titre in paired serum samples if diagnosed by IFA.

AKIN: Acute Kidney Injury Network, ELISA: Enzyme Linked Immunosorbent Assay, IgM: Immunoglobulin M.

Renal abnormalities were present in 82% cases, and >50% patients had AKI. Abnormal urinalysis was seen in 61% of patients. In a study from Vietnam, abnormal urinalysis, almost exclusively proteinuria, was reported in 20% of patients with scrub typhus diagnosed by IFA or mouse inoculation methods [38]. Mild proteinuria or microscopic hematuria have been reported in scrub typhus patients with acute renal failure [39]. A recent study from India reported abnormal urinalysis in 57% patients [36]. Low-grade albuminuria, along with microscopic hematuria, pyuria and glucosuria suggest predominant tubulo-interstitial involvement. Clinical evidence of glomerular involvement was seen in 2 cases, one as nephrotic range proteinuria that improved spontaneously, and the other with glomerular hematuria who was shown to have IgA nephropathy on biopsy. It is likely that concurrent febrile illness due to scrub typhus precipitated episode of gross hematuria in the second patient.

Other descriptions of acute renal failure in scrub typhus are in the form of case reports [39]–[43] and one autopsy study [44]. The mechanism of AKI in scrub typhus is mainly believed to be impaired renal perfusion due to volume depletion or increased vascular permeability [42]. Other potential mechanisms include direct tubular toxicity leading to acute tubular necrosis, interstitial nephritis, pigment nephropathy due to rhabdomyolysis and thrombotic microangiopathy secondary to disseminated intravascular coagulation. Renal biopsies have shown mild mesangial hyperplasia, acute tubular necrosis or tubulointerstitial nephritis [40], [42], [45]. The autopsy series published 67 years back described renal histopathologic changes in 69 patients with scrub typhus [44]. Proximal tubular epithelial swelling and interstitial nephritis were universal. Focal or diffuse glomerulonephritis was seen in 30% of kidney specimens. Fibrin filled capillaries or swollen endothelial cells leading to glomerular ischemia was another prominent observation. Kidneys were the second most common site of vascular changes after testes. Evidently, these findings represented involvement in severest form which led to death of patients.

In our study, the outcome of AKI was largely favourable as all the surviving patients with AKI had completely recovered their renal function. All 3 patients who had dialysis dependent AKI expired. This may be a reflection of underlying severity of disease. The significant association of hyperbilirubinemia with AKI and association of increased mortality with oliguric form of AKI may also be a reflection of the same. Basu et al [27] also showed increasing mortality risk with increasing severity of AKI.

There are some limitations to this study. Our study population is unlikely to represent the true burden of this disease as only those patients who had severe disease or were unresponsive to treatment in community health facilities would have been referred to our tertiary care centre. Secondly, the diagnosis was based on a single nucleic acid test. Other nucleic acid-based tests such as 47-kDa-based and GroEL-based real-time PCR and loop-mediated isothermal PCR [20] have also been used. It is possible that a combination of these along with culture and properly validated local serological criteria would allow identification of more cases.

Despite these limitation, our current observations are significant as they identify scrub typhus as an important cause of unexplained acute febrile illness in this region and contradict the current notion that AKI is uncommon complication in this disease. Whether the higher incidence of AKI in hospitalized patients with scrub typhus in our region compared to that reported from other centres is actually related to some host factor, differences in microbial virulence or is an actual unmasking of a previously unrecognized complication is difficult to infer due to variability and paucity of existing literature. Standardization of diagnostic criteria and uniform definitions of various manifestations when applied across various centres may help in this regard.

In conclusion, scrub typhus is an important cause of febrile illness with multisystem involvement in tropical regions. Renal involvement is common and AKI is an independent predictor of mortality. We suggest that the panel of investigation in all these cases should include PCR for O *tsutsugamushi*. The discrepancy between NAT positivity and IgM ELISA positivity as defined by arbitrary cutoffs suggests the need to develop robust local seroprevalence data.

## Supporting Information

Checklist S1 **STROBE checklist.**
(DOC)

## References

[1] WHO Recommended Surveillance Standards WHO/CDS/CSR/ISR/99.2. Second ed: World Health Organization. Available from: http://www.who.int/csr/resources/publications/surveillance/whocdscsrisr992syn.pdf.

[2] Watt G, Parola P (2003) Scrub typhus and tropical rickettsioses. Curr Opin Infect Dis 16: 429–436. 14501995 10.1097/00001432-200310000-00009

[3] Sharma A, Mahajan S, Gupta ML, Kanga A, Sharma V (2005) Investigation of an outbreak of scrub typhus in the himalayan region of India. Jpn J Infect Dis 58: 208–210. 16116251

[4] Vivekanandan M, Mani A, Priya YS, Singh AP, Jayakumar S, et al (2010) Outbreak of scrub typhus in Pondicherry. J Assoc Physicians India 58: 24–28. 20649095

[5] Khan SA, Dutta P, Khan AM, Topno R, Borah J, et al (2012) Re-emergence of scrub typhus in northeast India. Int J Infect Dis 16: e889–890. 22796321 10.1016/j.ijid.2012.05.1030

[6] Chrispal A, Boorugu H, Gopinath KG, Prakash JA, Chandy S, et al (2010) Scrub typhus: an unrecognized threat in South India - clinical profile and predictors of mortality. Trop Doct 40: 129–133. 20360426 10.1258/td.2010.090452

[7] Varghese GM, Abraham OC, Mathai D, Thomas K, Aaron R, et al (2006) Scrub typhus among hospitalised patients with febrile illness in South India: magnitude and clinical predictors. J Infect 52: 56–60. 16368461 10.1016/j.jinf.2005.02.001

[8] Vikrant S, Dheer SK, Parashar A, Gupta D, Thakur S, et al (2013) Scrub typhus associated acute kidney injury—a study from a tertiary care hospital from western Himalayan state of India. Renal Failure 1–6. 10.3109/0886022X.2013.828257 23952649

[9] John TJ, Dandona L, Sharma VP, Kakkar M (2011) Continuing challenge of infectious diseases in India. Lancet 377: 252–269. 21227500 10.1016/S0140-6736(10)61265-2

[10] Raoult D (2009) *Orientia tsutsugamushi* (Scrub Typhus). In: Mandell GL, Bennett JE, Dolin R, editors. Mandell: Mandell, Douglas, and Bennett's Principles and Practice of Infectious Diseases. Seventh ed: Churchill Livingstone, An Imprint of Elsevier. pp. 2529–2530.

[11] Reller ME, Dumler JS (2011) Scrub Typhus (*Orientia tsutsugamushi*). In: Kliegman RM, Stanton BF, GemeIII JWS, Schor NF, Behrman RE, editors. Kliegman: Nelson Textbook of Pediatrics. Nineteenth ed: Saunders, An Imprint of Elsevier. pp. 1045–1046.

[12] Chogle AR (2010) Diagnosis and treatment of scrub typhus–the Indian scenario. J Assoc Physicians India 58: 11–12. 20649092

[13] Saisongkorh W, Chenchittikul M, Silpapojakul K (2004) Evaluation of nested PCR for the diagnosis of scrub typhus among patients with acute pyrexia of unknown origin. Trans R Soc Trop Med Hyg 98: 360–366. 15099992 10.1016/j.trstmh.2003.10.012

[14] Kim DM, Park G, Kim HS, Lee JY, Neupane GP, et al (2011) Comparison of conventional, nested, and real-time quantitative PCR for diagnosis of scrub typhus. Journal of clinical microbiology 49: 607–612. 21068287 10.1128/JCM.01216-09 PMC3043474

[15] Richards AL (2012) Worldwide detection and identification of new and old rickettsiae and rickettsial diseases. FEMS Immunol Med Microbiol 64: 107–110. 22067055 10.1111/j.1574-695X.2011.00875.x

[16] Furuya Y, Yoshida Y, Katayama T, Yamamoto S, Kawamura A Jr (1993) Serotype-specific amplification of *Rickettsia tsutsugamushi* DNA by nested polymerase chain reaction. Journal of clinical microbiology 31: 1637–1640. 8315007 10.1128/jcm.31.6.1637-1640.1993 PMC265595

[17] Ranieri VM, Rubenfeld GD, Thompson BT, Ferguson ND, Caldwell E, et al (2012) Acute respiratory distress syndrome: the Berlin Definition. JAMA 307: 2526–2533. 22797452 10.1001/jama.2012.5669

[18] Kidney Disease: Improving Global Outcomes (KDIGO) Acute Kidney Injury Work Group. KDIGO Clinical Practice Guideline for Acute Kidney Injury. Kidney inter., Suppl. 2012; 2: : 1–138

[19] Kwok S, Higuchi R (1989) Avoiding false positives with PCR. Nature 339: 237–238. 2716852 10.1038/339237a0

[20] Blacksell SD, Bryant NJ, Paris DH, Doust JA, Sakoda Y, et al (2007) Scrub typhus serologic testing with the indirect immunofluorescence method as a diagnostic gold standard: a lack of consensus leads to a lot of confusion. Clin Infect Dis 44: 391–401. 17205447 10.1086/510585

[21] Paris DH, Blacksell SD, Nawtaisong P, Jenjaroen K, Teeraratkul A, et al (2011) Diagnostic accuracy of a loop-mediated isothermal PCR assay for detection of Orientia tsutsugamushi during acute Scrub Typhus infection. PLoS Neglected Tropical Diseases 5: e1307. 21931873 10.1371/journal.pntd.0001307 PMC3172190

[22] Koh GC, Maude RJ, Paris DH, Newton PN, Blacksell SD (2010) Diagnosis of scrub typhus. The American journal of tropical medicine and hygiene 82: 368–370. 20207857 10.4269/ajtmh.2010.09-0233 PMC2829893

[23] Paris DH, Shelite TR, Day NP, Walker DH (2013) Unresolved problems related to scrub typhus: a seriously neglected life-threatening disease. The American journal of tropical medicine and hygiene 89: 301–307. 23926142 10.4269/ajtmh.13-0064 PMC3741252

[24] Furuya Y, Yoshida Y, Katayama T, Kawamori F, Yamamoto S, et al (1991) Specific amplification of *Rickettsia tsutsugamushi* DNA from clinical specimens by polymerase chain reaction. Journal of clinical microbiology 29: 2628–2630. 1774275 10.1128/jcm.29.11.2628-2630.1991 PMC270390

[25] Ohashi N, Nashimoto H, Ikeda H, Tamura A (1990) Cloning and sequencing of the gene (tsg56) encoding a type-specific antigen from *Rickettsia tsutsugamushi* . Gene 91: 119–122. 2401407 10.1016/0378-1119(90)90171-m

[26] Kim DM, Yun NR, Yang TY, Lee JH, Yang JT, et al (2006) Usefulness of nested PCR for the diagnosis of scrub typhus in clinical practice: A prospective study. The American journal of tropical medicine and hygiene 75: 542–545. 16968938

[27] Basu G, Chrispal A, Boorugu H, Gopinath KG, Chandy S, et al (2011) Acute kidney injury in tropical acute febrile illness in a tertiary care centre–RIFLE criteria validation. Nephrol Dial Transplant 26: 524–531. 20702532 10.1093/ndt/gfq477

[28] Li PK-T, Burdmann EA, Mehta RL (2013) Acute kidney injury: Acute kidney injury[mdash]global health alert. Nat Rev Nephrol [epub ahead of print]. 10.1038/nrneph.2013.20 23399583

[29] Wang YC, Chen PC, Lee KF, Wu YC, Chiu CH (2013) Scrub typhus cases in a teaching hospital in Penghu, Taiwan, 2006–2010. Vector Borne Zoonotic Dis 13: 154–159. 23421889 10.1089/vbz.2012.1059

[30] Tilak R, Kunwar R, Wankhade UB, Tilak VW (2011) Emergence of Schoengastiella ligula as the vector of scrub typhus outbreak in Darjeeling: has Leptotrombidium deliense been replaced? Indian J Public Health 55: 92–99. 21941043 10.4103/0019-557X.85239

[31] Chandy SJ, Thomas K, Mathai E, Antonisamy B, Holloway KA, et al (2013) Patterns of antibiotic use in the community and challenges of antibiotic surveillance in a lower-middle-income country setting: a repeated cross-sectional study in Vellore, South India. J Antimicrob Chemother 68: 229–236. 22945913 10.1093/jac/dks355

[32] Kotwani A, Holloway K (2011) Trends in antibiotic use among outpatients in New Delhi, India. BMC Infect Dis 11: 99. 21507212 10.1186/1471-2334-11-99 PMC3097160

[33] Rajapakse S, Rodrigo C, Fernando D (2012) Scrub typhus: pathophysiology, clinical manifestations and prognosis. Asian Pac J Trop Med 5: 261–264. 22449515 10.1016/S1995-7645(12)60036-4

[34] Wu K-M, Wu Z-W, Peng G-Q, Wu JL, Lee S-Y (2009) Radiologic Pulmonary Findings, Clinical Manifestations and Serious Complications in Scrub Typhus: Experiences From A Teaching Hospital in Eastern Taiwan. International Journal of Gerontology 3: 223–232.

[35] Tsay RW, Chang FY (1998) Serious complications in scrub typhus. J Microbiol Immunol Infect 31: 240–244. 10496165

[36] Attur RP, Kuppasamy S, Bairy M, Nagaraju SP, Pammidi NR, et al (2013) Acute kidney injury in scrub typhus. Clin Exp Nephrol 17 725–729. 23292176 10.1007/s10157-012-0753-9

[37] Kumar M, Krishnamurthy S, Delhikumar CG, Narayanan P, Biswal N, et al (2012) Scrub typhus in children at a tertiary hospital in southern India: clinical profile and complications. J Infect Public Health 5: 82–88. 22341847 10.1016/j.jiph.2011.11.001

[38] Berman SJ, Kundin WD (1973) Scrub typhus in South Vietnam. A study of 87 cases. Ann Intern Med 79: 26–30. 4198459 10.7326/0003-4819-79-1-26

[39] Yen TH, Chang CT, Lin JL, Jiang JR, Lee KF (2003) Scrub typhus: a frequently overlooked cause of acute renal failure. Ren Fail 25: 397–410. 12803503 10.1081/jdi-120021152

[40] Hsu GJ, Young T, Peng MY, Chang FY, Chou MY, et al (1993) Acute renal failure associated with scrub typhus: report of a case. J Formos Med Assoc 92: 475–477. 8104604

[41] Lee S, Kang KP, Kim W, Kang SK, Lee HB, et al (2003) A case of acute renal failure, rhabdomyolysis and disseminated intravascular coagulation associated with scrub typhus. Clin Nephrol 60: 59–61. 12872861 10.5414/cnp60059

[42] Kim DM, Kang DW, Kim JO, Chung JH, Kim HL, et al (2008) Acute renal failure due to acute tubular necrosis caused by direct invasion of *Orientia tsutsugamushi* . Journal of clinical microbiology 46: 1548–1550. 18003808 10.1128/JCM.01040-07 PMC2292935

[43] Young PC, Hae CC, Lee KH, Hoon CJ (2003) Tsutsugamushi infection-associated acute rhabdomyolysis and acute renal failure. Korean J Intern Med 18: 248–250. 14717236 10.3904/kjim.2003.18.4.248 PMC4531640

[44] Allen AC, Spitz S (1945) A Comparative Study of the Pathology of Scrub Typhus (Tsutsugamushi Disease) and Other Rickettsial Diseases. Am J Pathol 21: 603–681. 19970829 PMC1934171

[45] Chi WC, Huang JJ, Sung JM, Lan RR, Ko WC, et al (1997) Scrub typhus associated with multiorgan failure: a case report. Scand J Infect Dis 29: 634–635. 9571750 10.3109/00365549709035911

